# Guiding prosthetic femoral version using 3D-printed patient-specific instrumentation (PSI): a pilot study

**DOI:** 10.1186/s41205-023-00168-w

**Published:** 2023-04-14

**Authors:** Maria Moralidou, Johann Henckel, Anna Di Laura, Alister Hart

**Affiliations:** 1 Institute of Orthopaedics and Musculoskeletal Science, University College London, Stanmore, UK; 2 Royal National Orthopaedic Hospital NHS Trust, Stanmore, UK; 3 Department of Mechanical Engineering, University College London, London, UK

**Keywords:** Primary total hip arthroplasty, Prosthetic femoral version, 3D-Printed patient-specific guides

## Abstract

**Background:**

Implantation of the femoral component with suboptimal version is associated with instability of the reconstructed hip joint. High variability of Prosthetic Femoral Version (PFV) has been reported in primary Total Hip Arthroplasty (THA). Three-dimensional (3D) Patient-Specific Instrumentation (PSI) has been recently developed and may assist in delivering a PFV within the intended range.

We performed a pilot study to better understand whether the intra-operative use of a novel PSI guide, designed to deliver a PFV of 20°, results in the target range of PFV in primary cemented THA.

**Methods:**

We analysed post-operative Computed-Tomography (CT) data of two groups of patients who underwent primary cemented THA through posterior approach; 1. A group of 11 patients (11 hips) for which the surgeon used an intra-operative 3D-printed stem positioning guide (experimental) 2. A group of 24 patients (25 hips) for which the surgeon did not use the guide (control). The surgeon aimed for a PFV of 20°, and therefore the guide was designed to indicate the angle at which the stem was positioned intra-operatively. PFV angles were measured using the post-operative 3D-CT models of the proximal femurs and prosthetic components in both groups. Our primary objective was to compare the PFV in both groups. Our secondary objective was to evaluate the clinical outcome.

**Results:**

Mean (± SD) values for the PFV was 21.3° (± 4.6°) and 24.6° (± 8.2°) for the experimental and control groups respectively. In the control group, 20% of the patients reported a PFV outside the intended range of 10° to 30° anteversion. In the experimental group, this percentage dropped to 0%. Satisfactory clinical outcome was recorded in both groups.

**Conclusion:**

The intra-operative use of a PSI PFV guide helped the surgeon avoid suboptimal PFV in primary cemented THA. Further studies are needed to evaluate if the PSI guide directly contributes to a better clinical outcome.

## Background

Implantation of the femoral component with suboptimal Prosthetic Femoral Version (PFV) is associated with rotational instability [1], elevated torsional moments [2], impingement [3] and dislocation [4, 5] in primary Total Hip Arthroplasty (THA).

Previous studies have highlighted the high variability of PFV in primary uncemented THA, with post-operative values ranging from -23° to 39° [6–8]. The final PFV of an uncemented straight femoral stem is a result of press-fitting of an object of a pre-defined geometrical shape (the stem) into a highly irregular anatomical space (the proximal femur), leaving the surgeon with limited control over its final orientation [9–12]. Pre-operative Two-Dimensional (2D) radiographic analysis of the intended PFV is not applicable either; the native version of the proximal femur (known as NFV) significantly deviates from the PFV of an uncemented femoral stem and is not a reliable reference [11–15].

The malleable nature of the cement mantle in the cemented fixation allows the surgeon to adjust the PFV independent of the anatomy of the intramedullary canal, and may be considered a targeted approach to deliver an adequate PFV [3, 9, 13, 16]. However, the surgeon visually assesses the PFV of a cemented femoral stem through knee flexion and vertical placement of the leg [17]. This technique has proven to be imprecise [16, 18], indicating the need to intra-operatively guide the PFV of the femoral stem.

Three-Dimensional (3D) Patient-Specific Instrumentation (PSI) has been recently developed to guide the implantation of the femoral component in primary THA [19, 20] and may act as an aiding tool in guiding the PFV within the intended range.

We aimed to better understand whether the intra-operative use of a novel PSI guide, designed to deliver a PFV of 20°, results in the delivery of the target (surgical target ± 10°) in primary THA. Our primary objective was to compare the PFV in two THA groups; 1. An experimental group for which the surgeon used the guide; 2. A control group for which the surgeon did not use the guide. Our secondary objective was to evaluate the clinical outcome.

## Methods

### Study design

**Fig. 1:**
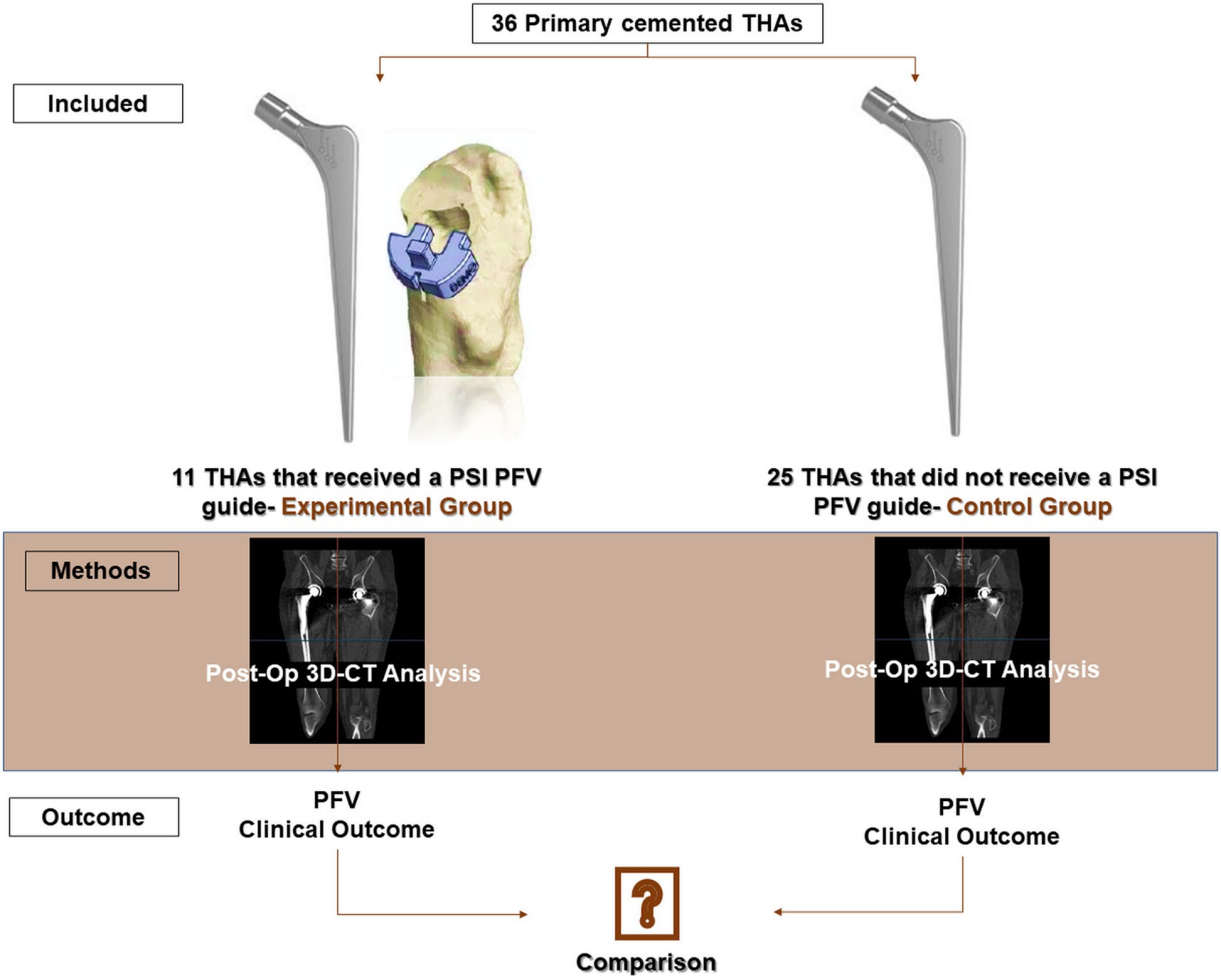
Study design

We performed a pilot study involving a total of 35 patients (36 hips) undergoing primary cemented THA due to osteoarthritis (OA), between February 2020 and December 2021. Two groups of patients were studied; first, one group of 11 patients (11 hips) and a second group  group of 24 patients (25 hips) who underwent PSI-guided (experimental group) and non-guided (control group) primary cemented THA respectively. A PSI PFV guide was designed and 3D-printed for each case, designed on the pre-operative CT data. The PSI PFV guide was intra-operatively used to guide the PFV in the experimental group. PFV angles were subsequently measured based on the post-operative 3D-CT data in both groups (Fig. 1 and Table 1).

**Table 1:** Study groups characteristics

	**Experimental Group (*N* = 11 Hips)**	**Control Group (*N* = 25 Hips)**	***P* Value**
Gender (Females) (%)	8 (73)	14 (56)	0.35
Age (Years) (Median, Range)	69 (48–83)	64 (42–89)	0.64
Treatment Side (Right) (%)	9 (82)	17 (68)	0.4

The outcome measures were:

- PFV angles.
- Clinical outcome.

### Pre-operative scanning and surgical planning

Prior to the surgery, all patients underwent CT scanning of the hip and the knee joint for surgical planning, according to a standard protocol (Somatom Definition AS, Siemens, Germany). Image acquisition consisted of two scans: 1. A scan of the pelvis and the proximal femur (10 cm below the Lesser Trochanter—LT); 2. A scan of the distal femur including the femoral condyles [21].

All patients underwent pre-operative CT planning using proprietary software (MyHip Planner, Medacta International SA, Castel San Pietro, Switzerland) to choose type, size and position of the femoral and acetabular components. Selection of the femoral head component was done to reconstruct the Femoral Offset (FO) and of the femoral stem to maximize the contact between the bone and the implant [21]. The femoral neck osteotomy plane was defined with reference to the contralateral side, to restore the leg length. Osteotomy angle was planned at 45° relative to the long axis of the proximal femur.

**Fig. 2:**
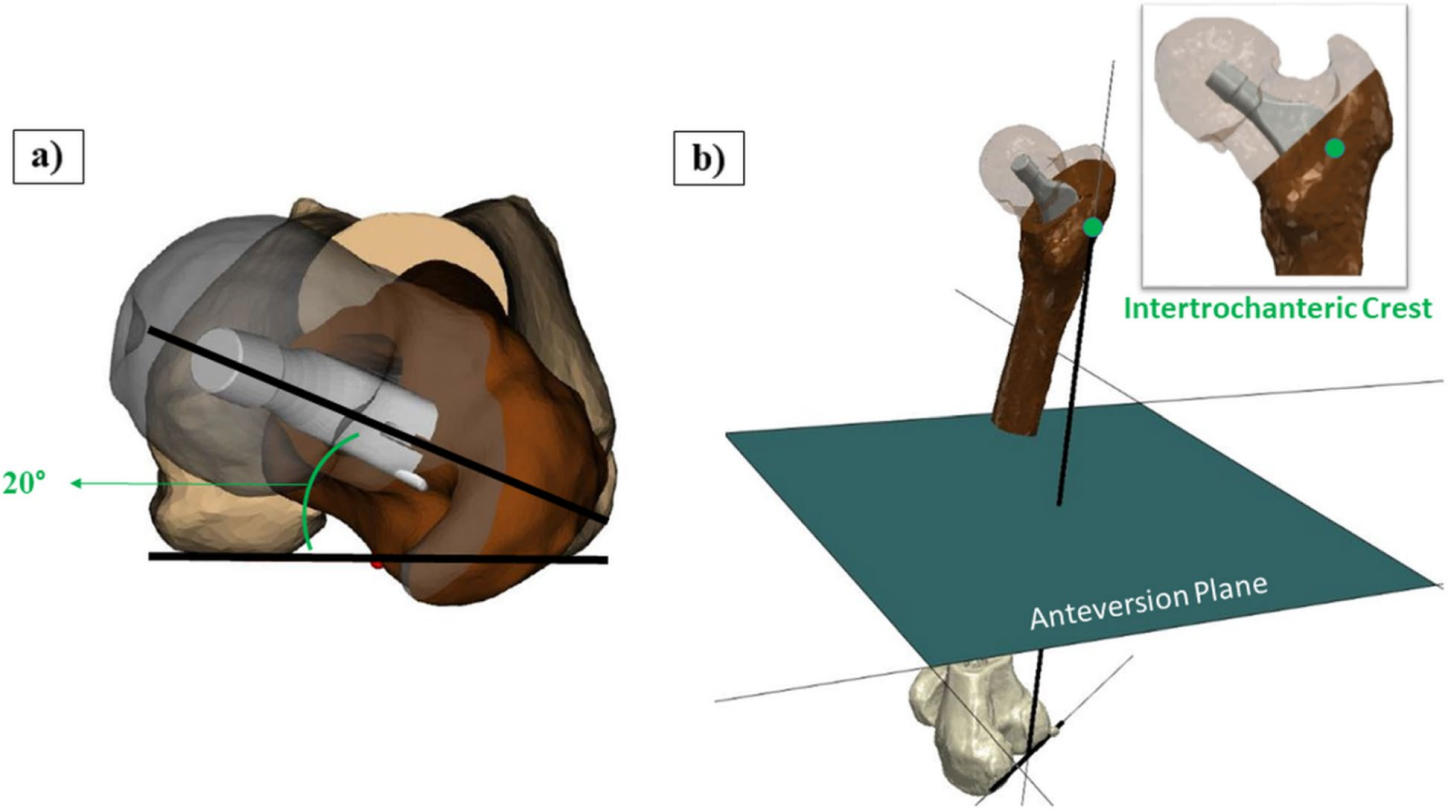
**a** PFV; **b** Coordinate system used to plan and measure PFV

PFV was planned at an angle of 20° relative to the Posterior Condylar Axis (PCA) (Fig. 2a), as measured on a plane perpendicular to the axis connecting the midpoint of the PCA and the intertrochanteric crest (a bone eminence located at the posterior aspect of the proximal femur) (Fig. 2b). Regarding the acetabular angles, cup inclination and anteversion angles were planned at 40° and 20°, respectively.

**Fig. 3:**
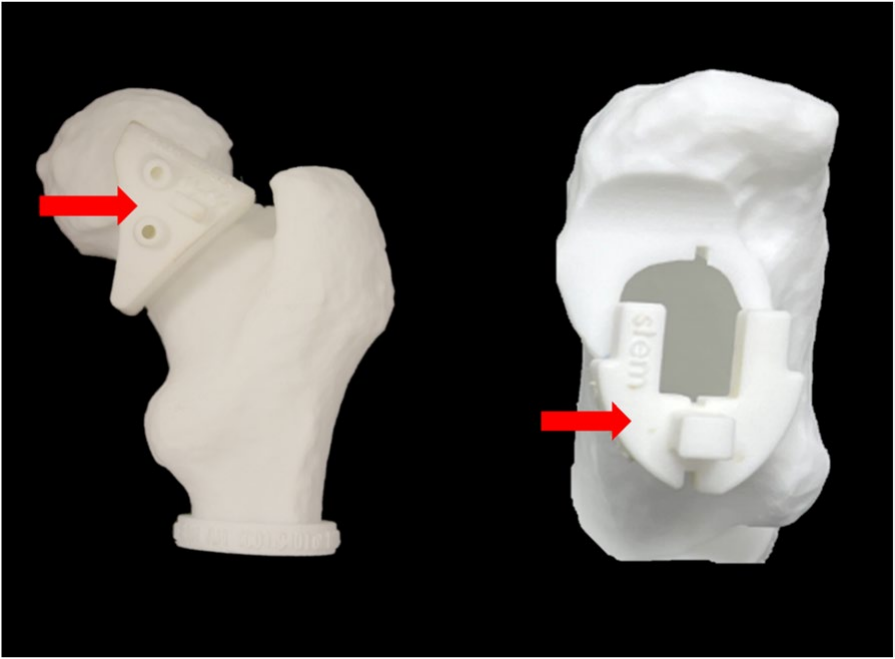
Descriptive illustration of the two PSI guides that were used: **a** To perform the femoral neck osteotomy; **b** To guide the PFV at an angle of 20°

The surgical plan included two PSI guides; 1. To define the osteotomy level and angle, 2. To guide PFV at an angle of 20°. The PSI femoral neck osteotomy guide was 3D-printed to perfectly fit the contours of the femoral head-neck junction and deliver the planned osteotomy plane. The PSI PFV guide was designed to include 3D-printed incorporated slots, indicating a PFV of 20°. (Fig. 3).

The PSI guides were 3D-printed using an EOS Selective Laser Sintering (SLS) printer. (EOS, Munich, Germany). The printing material was Polyamide (PA) 2200. The PSI guides were then sterilised using gamma irradiation or an autoclave and shipped according to current standards to the hospital before the surgical use.

### Surgical approach, prosthetic components and PSI

A single consultant orthopaedic surgeon performed all the surgeries through a posterior approach. A collarless double-tapered femoral stem (X-Acta system; Medacta International SA, Castel san Pietro, Switzerland) and a hemispheric Hydroxyapatite (HA) coated cup were used (Mpact system; Medacta International SA, Castel san Pietro, Switzerland).

Intra-operatively, the sterilised PSI osteotomy guide was placed on the femoral neck and two pins secured its fitting. The femoral neck osteotomy was then performed with the oscillating saw blade flush on the flat side of the PSI cutting jig. In the experimental group, the sterilised 3D-printed PSI PFV guide was secured on the osteotomy plane. The surgeon then adjusted the PFV of the femoral stem to be aligned to the indicated slots. In the control group, the surgeon visually adjusted the femoral stem using the cement mantle to deliver a PFV of 20°.

### Post-operative scanning

All patients underwent post-operative CT scanning of the hip region and the knee joint using the same scanning protocol adopted for the scans acquired before the surgery. The scans were corrected for metal artefacts; Normalized Metal-Artefact Reduction (NMAR) algorithm was implemented to the post-operative CT scans. 3D models of the post-operative femurs and prosthetic components were subsequently generated, using Simpleware ScanIP software (Version 2021.03; Synopsys, Inc., Mountain View, USA).

### Measurement of PFV

PFV was measured; the angle between the neck of the reconstructed femur and the PCA. The stem neck axis was defined as the line connecting the centre of the head with the top mark of the stem [6]. The measurements were taken using the same coordinate system that was adopted during pre-operative planning.

### Statistical analysis

Statistical analysis software (SPSS, version 28, Chicago, USA) was used to compute the descriptive statistics for the outcome measures. In order to establish whether data analysed in this study was normally distributed, the Shapiro–Wilk test (n < 50) was utilized. The Mann–Whitney U test was implemented to evaluate differences between the two groups with regard to the study group characteristics.

## Results

### PFV

#### Comparison of PFV in experimental and control groups

**Fig. 4:**
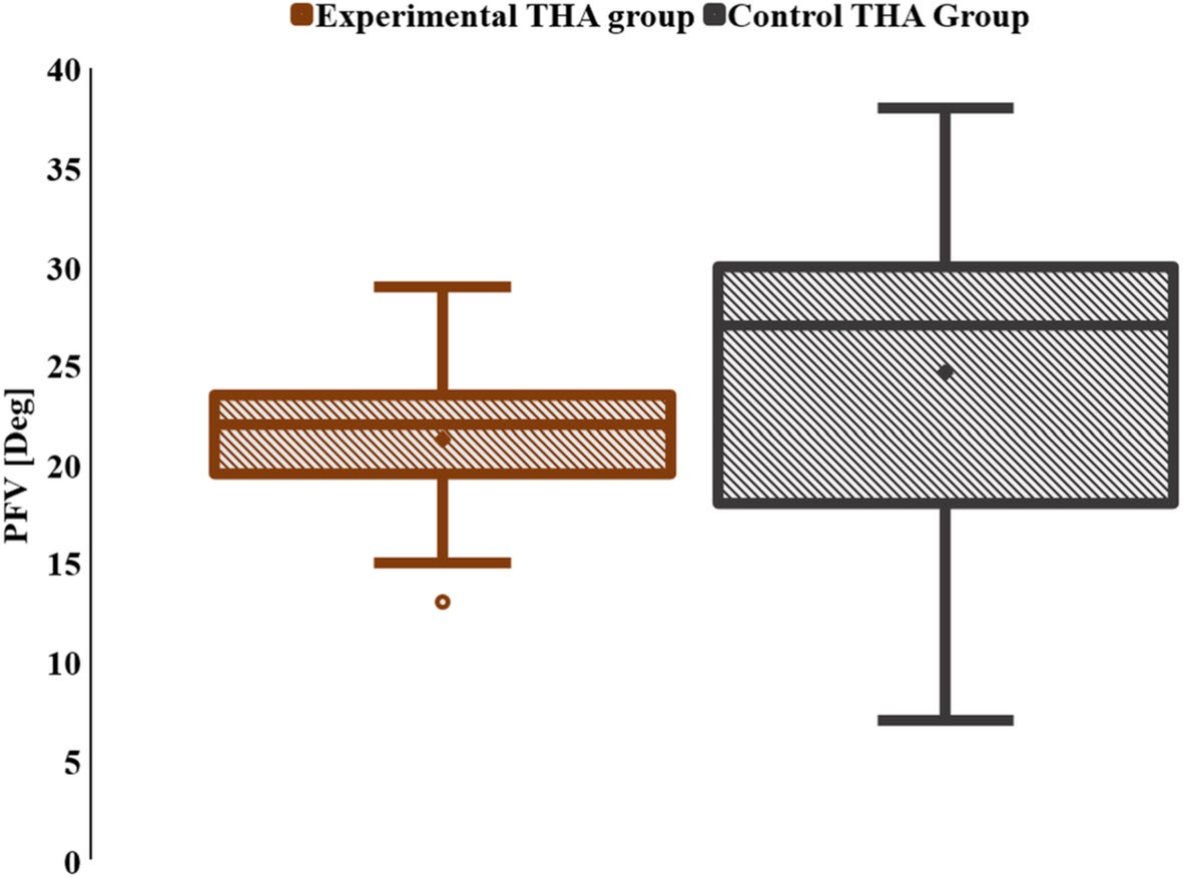
Box plot illustrating the PFV in experimental and control THA groups

The data describing the PFV angles in the experimental and control groups matched the tendency expected for a normal distribution (Shapiro–Wilk, p1 = 0.85, p2 = 0.4). The experimental group had mean (± SD) and median (IQR) PFV of 21.3° (± 4.6°) and 22° (19.5–23.5°), respectively. The control group, had a mean (± SD) and median (IQR) PFV of 24.6° (± 8.2°) and 27° (18–30°), respectively. (Fig. 4).

##### Distribution of PFV in experimental and control groups

**Fig. 5:**
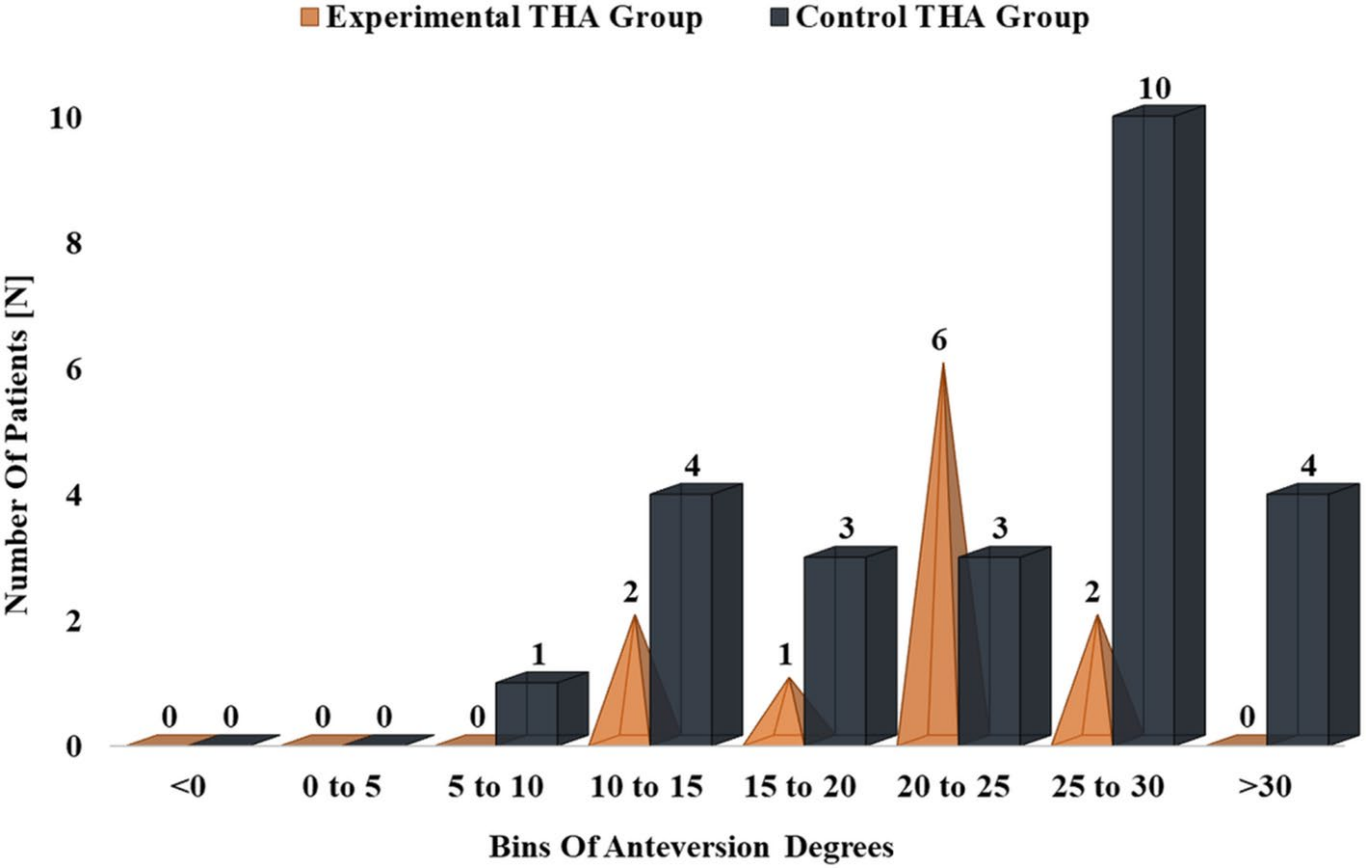
Histogram depicting the distribution of PFV in experimental and control THA groups

In the experimental group, 18% of the femoral stems reported a PFV between 10° and 15°. A PFV of between 15° and 20° and between 20° and 25° was reported in 9% and 55% of the femoral stems respectively. Eighteen per cent (18%) of the femoral stems reported a PFV between 25° and 30°. (Fig. 5).

 Regarding the distribution of PFV in the control group, a PFV of between 5° and 10° and between 10° and 15° was reported in 4% and 16% of the femoral stems respectively. Twelve per cent (12%) of the femoral stems had a PFV of between 15° and 20° and between 20° and 25°. Finally, 40% of the femoral stems were anteverted of between 25° and 30° and 16% were anteverted of more than 30°. (Fig. 5).

### Clinical outcomes

The experimental group had a median follow up time of 11 months (10 to 13 months). The median follow-up time for the control group was 23 months (16 to 32 months). No intra-operative complications such as fracture, indicating wrong implant planning, size or implantation, have been recorded, resulting in an overall functional clinical outcome. At the most recent follow-up, none of the hips had been revised for any reason. Post-operative evaluation revealed adequate fixation with no loosening occurring at one year after the surgery.

## Discussion

This was the first study to assess the intra-operative use of a 3D-printed PSI guide, engineered to deliver a PFV of 20° in primary cemented THA using 3D-CT analysis. PFV angles were measured in two groups of patients: 1. A group of THA patients for which the surgeon used a PSI PFV guide (experimental group); 2. A group of THA patients for which the surgeon did use the PSI PFV guide (control group). We found that through the use of the PSI PFV guide, a lower variability of PFV was reported in the experimental group, when compared to the control group.

PFV constitutes a critical variable associated with the biomechanical stability of the hip joint [1, 2, 4, 5, 22]. Existing literature has reported a high variability of PFV in primary uncemented THA, ranging from -23° to 39° [6–8]. In the cemented THA, the surgeon can adjust the orientation of the femoral stem to match his intra-operative estimation [3, 9, 13]. Previous studies, however, have proved that visual intra-operative estimation of the PFV is imprecise [16, 18]. The results of this study confirmed this. The PFV of a non-guided cemented femoral stem ranged between 7° and 38°.

The high variability of PFV in primary THA indicated the need to guide the femoral component orientation within the intended range. Although the optimal range for the acetabular cup version has been well established, little has been published about the optimal range for PFV. Dorr et al. (2009) have reported that the generally accepted range of PFV is between 10° and 20° [16], while Reikeras et al. (2011) reported that the intended range of PFV is between 10° and 30° [23]. Additionally, it has been reported that there is no consensus among surgeons regarding the optimal goal for PFV [24].

A normal PFV is typically assumed between 15° and 20° [25]. Since a low PFV has been linked to dislocation in primary THA with a posterior approach [4, 5], the surgical target of PFV in this single-surgeon series was set at the top of what is considered to be the normal range (surgical target = 20°). According to the results of this study, 20% of the patients reported a PFV outside the intended range (surgical target ± 10°) in the control group. This percentage dropped to 0% in the experimental group.

**Fig. 6:**
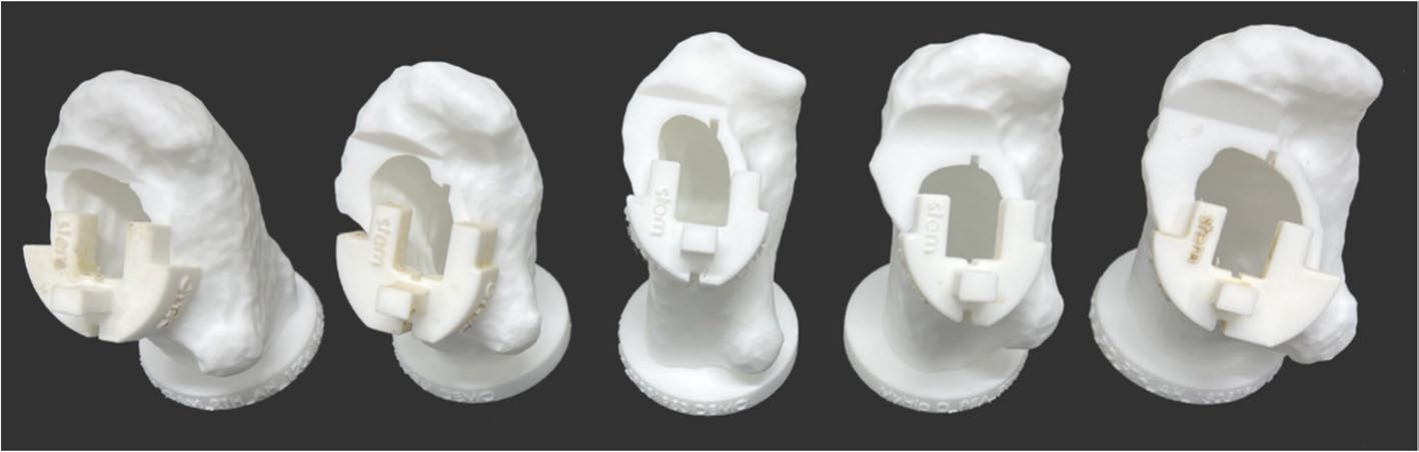
3D-Printed models of the proximal femurs and of the PSI guides with incorporated slots indicating the target of the PFV that were used intra-operatively

Figure 6 illustrates five of the 3D-printed femoral bony models and the PSI PFV guides that were used during the surgery. Visual intra-operative adjustment of the femoral stem is necessary to align the femoral stem neck axis according to the slots. Incorporating the PSI PFV guide with an uncemented femoral stem would be ineffective. The orientation of an uncemented femoral stem is dictated by the internal morphology of the proximal femur and the surgeon cannot fully control the final PFV [11, 12]. For this reason, we used a cemented double-tapered highly polished femoral stem design, due to the intra-operative adjustability that it has been observed to surgically offer.

Given that there is a constant debate around the choice of component fixation in hip arthroplasty, the surgical use of the specific PSI guide may restrict the surgical application of uncemented fixation, which is preferred when chronological and bone assessment criteria are considered [26]. However, considering the importance of an adequate PFV and how this affects the biomechanics of the hip joint, PFV may constitute a further criterion in selecting the most appropriate fixation technique.

We acknowledge limitations. First, this was a pilot study including a small number of cases. A prospective randomised controlled trial with a large number of cases should be conducted in the future to compare the accuracy of the PSI PFV guide to the usual technique. Second, a single design of a cemented femoral stem component was adopted. Third, all surgeries were performed through a posterior approach. Different surgical approaches may have an effect on the delivered PFV and the design of the guide. In addition, the fitting of this PSI guide is dependent on the accuracy of the PSI femoral neck osteotomy guide. Potential error induced by the osteotomy guide may impact the fitting of the PFV guide. In this study, the osteotomy level was defined using a commercially-available PSI cutting guide, that has been proved to deliver the femoral neck osteotomy with a high level of accuracy to the surgical plan [27].

As far as the methodology is concerned, the processing chain of the implemented method comprised automated steps that aim to eliminate the variability of the outcome measures. The main limitation of this method relies on the measurement error of the post-operative analysis due to the manual selection of bony landmarks, the metal artefact and the scanning procedure. In light of this, the CT scans were corrected for metal artefacts and the PFV angles were measured based on 3D-CT models using clearly identifiable marks and a standardised coordinate system, that is not affected by the patient’s position within the scanner. In addition, all patients in this study underwent post-operative CT scanning using a low-dose scanning protocol designed to minimise radiation exposure while preserving spatial accuracy.

The potential to minimise complications through correct implant orientation using 3D-printed guides instead of highly expensive robotic-assisted surgery [28] benefits all the players involved in the chain.

## Conclusions

Optimal PFV is considered critical to ensure biomechanical stability of the hip joint, normal post-operative gait and satisfactory clinical outcomes. Recent CT studies have reported a wide range of PFV delivered in primary THA. In this pilot study, a 3D-printed PSI guide was used to deliver a PFV of 20°. We found that through the use of the guide, the PFV of a cemented femoral stem was within the target range (surgical target ± 10°). The concept of PSI is still in its infancy and further studies are needed to evaluate if the PS guide directly contributes to a better clinical outcome.

## Data Availability

Measurement data presented in this study is available upon reasonable request.

## Abbreviations

CT: Computed-Tomography
FO: Femoral Offset
HA: Hydroxyapatite
IQR: Interquartile Range
LT: Lesser Trochanter
NFV: Native Femoral Version
NMAR: Normalized Metal-Artefact Reduction
PA: Polyamide
PCA: Posterior Condylar Axis
PFV: Prosthetic Femoral Version
PSI: Patient-Specific Instrumentation
SD: Standard Deviation
SLS: Selective Laser Sintering
3D-CT: Three-Dimensional Computed-Tomography
2D: Two-Dimensional
THA: Total Hip Arthroplasty
